# Lutein as a Modulator of Oxidative Stress-Mediated Inflammatory Diseases

**DOI:** 10.3390/antiox10091448

**Published:** 2021-09-13

**Authors:** Yu Jin Ahn, Hyeyoung Kim

**Affiliations:** Department of Food and Nutrition, BK21 FOUR, College of Human Ecology, Yonsei University, Seoul 03722, Korea; anyujin@gmail.com

**Keywords:** inflammation, lutein, reactive oxygen species

## Abstract

Lutein is a xanthophyll carotenoid obtained from various foods, such as dark green leafy vegetables and egg yolk. Lutein has antioxidant activity and scavenges reactive oxygen species such as singlet oxygen and lipid peroxy radicals. Oxidative stress activates inflammatory mediators, leading to the development of metabolic and inflammatory diseases. Thus, recent basic and clinical studies have investigated the anti-inflammatory effects of lutein based on its antioxidant activity and modulation of oxidant-sensitive inflammatory signaling pathways. Lutein suppresses activation of nuclear factor-kB and signal transducer and activator of transcription 3, and induction of inflammatory cytokines (interleukin-1β, interleukin-6, monocyte chemoattratant protein-1, tumor necrosis factor-α) and inflammatory enzymes (cyclooxygenase-2, inducible nitric oxide synthase). It also maintains the content of endogenous antioxidant (glutathione) and activates nuclear factor erythroid 2–related factor 2 (Nrf2) and Nrf2 signaling-related antioxidant enzymes (hemeoxygenase-1, NAD(P)H: quinone oxidoreductase 1, glutathione-s-transferase, glutathione peroxidase, superoxide dismutase, catalase). In this review, we have discussed the current knowledge regarding the anti-inflammatory function of lutein against inflammatory diseases in various organs, including neurodegenerative disorders, eye diseases, diabetic retinopathy, osteoporosis, cardiovascular diseases, skin diseases, liver injury, obesity, and colon diseases.

## 1. Introduction

Carotenoids are divided into two classes based on their chemical structure: the carotenes (hydrocarbons, such as β-carotene and lycopene) and xanthophylls (polar compounds that contain oxygen atoms in their molecules, such as lutein and its stereoisomer zeaxanthin) [1]. Lutein is the second most prevalent carotenoid in human serum and is synthesized only by plants. It is abundantly present in eggs and dark green leafy vegetables such as kale and spinach [2,3,4].

Lutein acts as an antioxidant and protects plants from photo-induced free radical damage [5]. Xanthophyll carotenoids modulate oxidative stress and regulate redox-sensitive intracellular signaling [6]. Ozawa et al. [7] suggested that lutein inhibited oxidative stress-induced triggering of inflammatory signaling pathways such as the activated signal transducer and activator of transcription 3 (STAT3) signaling pathway and IL-6 expression in the retina. Lutein preserves visual function by preventing degradation of the functional proteins, rhodopsin (a visual pigment) and synaptophysin (a synaptic vesicle protein that is altered in neurodegenerative diseases). Lutein treatment reduced the concentrations of nitric oxide (NO), tumor necrosis factor (TNF)-α, interleukin (IL)-6, prostaglandin (PG)E_2_, and monocyte chemoattractant protein (MCP)-1 in aqueous humor of mice with endotoxin-induced uveitis [8]. Lutein treatment suppressed the development of choroidal neovascularization, which plays a critical role in the pathogenesis of age-related macular degeneration and inflammatory processes, including nuclear factor (NF)-κB activation and subsequent upregulation of inflammatory molecules such as MCP-1 in mice [9]. Horvath et al. [10] found that lutein inhibited the activation of transient receptor potential ankyrin 1 and the resultant inflammation of the mouse skin. This study showed that lutein decreased TRPA1 activation-induced neutrophil accumulation. Although these significant findings provide new insights into the anti-inflammatory actions of lutein, the mechanism underlying these observations need to be further investigated in humans.

Lutein has a long carbon chain with alternating single and double carbon-carbon bonds with attached methyl side groups. Due to the presence of a hydroxyl group at both ends of the molecule, lutein has distinct characteristics compared to other carotenoids [4]. Anti-inflammatory and anti-oxidant effects of lutein are attributed to its unique structure, particularly the presence of conjugated double bonds and hydroxyl groups [11]. The conjugated double bond acts as a powerful antioxidant by donating the electrons and reacting with free radicals to form a more stable product. This structural feature may also affect its uptake efficiency via the modulation of carotenoid polarity and flexibility.

Lutein is mainly delivered to the retina; therefore, most studies have focused on its visual activity. Lutein has attracted attention in relation to human health due to its putative role in protection against other inflammatory diseases, in addition to eye diseases. Dietary guidance for lutein shows that it has antioxidant and anti-inflammatory effects [12]. This review covers the current understanding of the protective effects of lutein against oxidative stress-mediated inflammatory diseases.

### 1.1. Absorption and Transport of Lutein

Lutein is accumulated in the eyes, liver, and lipophilic tissues, such as adipose tissue. It is transported from the gut to various organs through the bloodstream via lipoproteins [13]. The polar and flexible structure of the lutein molecule increases the affinity for lipid transporters and plasma membranes, leading to its increased absorption in the gut [14,15,16]. Lutein uptake occurs by both simple and facilitated diffusion and is mediated through cholesterol membrane transporters such as scavenger receptor class B member 1 (SR-B1) and a cluster of differentiation 36 (CD 36) [17,18]. When lutein is emulsified into small lipid droplets or vesicles in the stomach, it is converted into mixed micelles by bile salts with biliary phospholipids. Then, these mixed micelles are taken up by enterocytes with SR-B1 [19].

### 1.2. Bioavailability and Metabolism of Lutein

The bioavailability of lutein is affected by food source and matrix, fat content, processing, cooking, and dietary fiber. Depending on solubilization in the digestive system, the bioavailability of lutein is about 10–15% [20,21], which is very poor. Lutein has poor oral absorption because the high hydrophobicity of the C40 isoprenoid carbon skeleton of lutein makes it soluble in digestive fluids [22]. Lutein and its metabolites are found in the liver, plasma, retina, and adipose tissue. The common metabolites of lutein are 3′-hydroxy-ε,ε-caroten-3-one, 3′-hydroxy-ε,ε-caroten-3-one, and 3-hydroxy-β,ε-caroten-3′-one. In the mice model, the first two were mostly found in the plasma, kidney, adipose tissue, and liver with lutein. However, 3-hydroxy-β,ε-caroten-3′ was the major metabolite of lutein in human retina and plasma [22].

### 1.3. Toxicity and Safety of Lutein

There have been no reports of adverse effects on the genotoxicity of lutein formulations. The upper limit of safe lutein consumption has been set to 20 mg/day [23]. The daily intake of lutein is 2 mg/kg body weight, which is equivalent to 120 mg/day for a 60 kg person. Furthermore, long-term supplementation of dietary lutein has not been shown to have any adverse effects in humans. These studies found higher doses of lutein (30 mg and 40 mg/kg body weight) [24,25] to be safe. Similarly, lutein did not show any safety concerns in rats and monkeys [26,27].

## 2. Lutein in Inflammatory Diseases

### 2.1. Neurodegenerative Disorders

Xanthophylls, such as lutein and zeaxanthin, cross the blood-retina barrier to form the macular pigment in the eye [28]. The lutein level in the macula was found to be significantly correlated with its concentration in matched brain tissue. A significant correlation was observed between macular pigment density and global cognitive function in healthy older adults [28]. Lutein, zeaxanthin, and meso-zeaxanthin are collectively called the macular pigment [29]. Adequate maternal intake of lutein couples with the placental transfer of maternal lutein to support fetal brain and retina development [29]. Therefore, macular pigment is used as a biomarker of lutein in brain tissues. A recent study suggested that lutein preferentially accumulated in those regions in the brain that are related to visual perception, cognition, and motor coordination [30].

**Figure 1:**
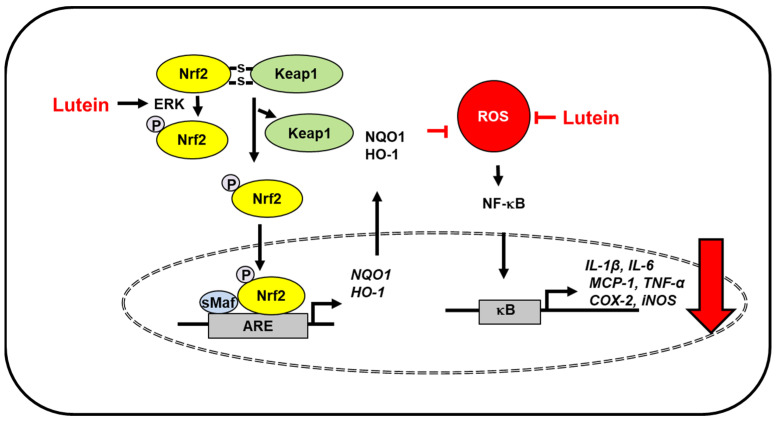
The proposed mechanism by which lutein inhibits oxidative stress-induced inflammatory responses in the brain. ROS levels increase in severe traumatic brain injury and lipopolysaccharide-activated microglia. Lutein reduces ROS levels and inhibits ROS-mediated activation of NF-kB and expression of inflammatory mediators (IL-1β, IL-6, MCP-1, TNF-α, COX-2, iNOS) [37]. In lipopolysaccharide-activated microglia, lutein activates ERK, which phosphorylates Nrf2 and increases dissociation of Keap1 from the Nfr2/Keap1 complex. Thus, it promotes nuclear translocation of Nrf2, which forms a heterodimer with sMaf protein and binds to a regulatory region of DNA called ARE. It induces the expression of Nrf2- target antioxidant genes (HO-1, NQO1). These antioxidant enzymes reduce intracellular ROS levels, which suppresses inflammatory responses [38]. Thus, lutein prevents oxidative stress-mediated neuroinflammation. ARE, antioxidant response element; COX-2, cyclooxygenase-2; ERK, extracellular signal-regulated kinase; HO-1, hemeoxygenase-1; iNOS, inducible nitric oxide synthase; IL, interleukin; Keap1, kelch like ECH associated protein 1; MCP-1; monocyte chemoattratant protein-1; NF−κB, nuclear factor-κB; Nrf2, nuclear factor erythroid 2–related factor 2; NQO-1, NAD(P)H: quinone oxidoreductase 1; ROS, reactive oxygen species; sMaf, small Maf; TNF-α, tumor necrosis factor-α.

Oxidative stress and inflammation of neural tissues induce age-related macular degeneration and Alzheimer’s disease. The Irish longitudinal study demonstrated the relationship between lutein, a plasma antioxidant, and improved cognitive function in healthy older adults [31]. Moreover, lutein depletion was observed in individuals with mild cognitive impairment [32] and Alzheimer’s disease [33,34]. A study using data from the third Nutrition and Health Examination Survey (NHANES III) database and the NHANES III Linked Mortality File suggested that high levels of lutein reduce the risk of mortality due to Alzheimer’s disease in older adults [35]. Severe traumatic brain injury is involved in oxidative stress-induced inflammation and apoptosis [36]. Lutein protected against severe traumatic brain injury by suppressing IL-1β, IL-6, MCP-1 expression and reducing serum reactive oxygen species (ROS) levels in rats with severe traumatic brain injury [37]. Additionally, lutein attenuated neuroinflammation in lipopolysaccharide-activated microglia by inhibiting inflammatory signaling such as NF-κB and expression of TNF-α, IL-1β, inducible NO synthase (iNOS), and cyclooxygenase-2 (COX-2). Lutein promoted nuclear factor erythroid 2–related factor 2 (Nrf2) activation and subsequent upregulation of heme oxygenase (HO)-1 and NAD(P)H: quinone oxidoreductase 1 (NQO1). The effect of lutein for Nrf2 activation was mediated with extracellular signal-regulated kinase (ERK) [38]. Mitogen activated protein kinases, including ERK phosphorylate Nrf2, in modulating the Nrf2-dependent antioxidant response [39]. Therefore, lutein may activate ERK, which phosphorylates Nrf2 to translocate into the nucleus and induces the expression of antioxidant enzymes. These results demonstrated that lutein induced the expression of Nrf2-target genes (antioxidant enzymes) and reduced the levels of inflammatory mediators to protect against inflammation-related neurodegenerative disorders (Figure 1).

There are two types of Nrf2 activators. Most Nrf2 inducers interact with cysteine residues of kelch like ECH-associated protein 1 (Keap1) by utilizing the electrophilic nature of the molecules and inactivating the Keap1 E3 ligase activity that targets Nrf2 for ubiquitin-dependent degradation. The other type of Nrf2 inducer is nonelectrophilic inducers, which interrupt the interaction between Keap1 and Nrf2 [40]. Lutein does not have electrophilic groups. Thus, lutein metabolites that possess electrophilic groups may react with Keap1. In another way, lutein may directly disturb the interaction between Keap1 and Nrf2. Further study should be performed to determine whether lutein metabolites are electrophiles to react with cysteine residues of Keap1.

Since ROS activate Nrf2 signaling and produce antioxidant enzyems as a defense mechansim in some cells [41], further detailed study is necessary to determine the mechanism of how lutein induces dissociation of Nrf2/Keap1 and increases nuclear translocation of Nrf2.

Shimazu et al. [42] suggested that lutein attenuated acute inflammation-induced nocifensive behavior and augmented nociceptive processing of spinal trigeminal nucleus caudalis and upper cervical dorsal horn neurons. These regions relay information to higher pain centers about the location and intensity of pain stimulus. This study supports lutein as a potential therapeutic agent to reduce or prevent acute trigeminal inflammatory pain. Overall, dietary lutein may be beneficial in maintaining cognitive health and protecting against inflammation-induced neurodegenerative diseases.

### 2.2. Eye Diseases

Lutein, as a component of macular pigment, protects the macula from photo-oxidative damage and enhances visual function [29]. Lutein is an ocular antioxidant that can quench both singlet oxygen and lipid peroxy radicals [43]. In addition, lutein inhibits activation of STAT3 and IL-6 expression in the retina [7]. Therefore, supplementation with lutein has been very effective for restoring ocular antioxidants of age-related maculopathy and AMD [44,45,46]

Oxidative stress is an important factor in the pathogenesis of age-related macular degeneration; thus, anti-oxidative stress is a good marker for the prevention or treatment of age-related macular degeneration. Lutein is a very effective quencher of singlet molecular oxygen and lipid peroxy radicals. However, lutein gets oxidized to its corresponding radical cations in the process. These cations must be reduced to regenerate the original carotenoids, which thus, allows its use as an antioxidant [47]. Lutein reduced ROS levels and suppressed apoptosis by reversing G2/M phase arrest through activation of cyclin-dependent kinase 1 and cell division cycle 25C in retinal pigment epithelial cells exposed to hydrogen peroxide [48]. Bian et al. [49] showed that lutein suppressed lipopolysaccharide-stimulated production of IL-6 and TNF-α in both retinal pigmental epithelial cells and macrophages isolated from the peritoneum of age-related macular degeneration model mice.

Lutein treatment reduced the light-induced increase in local ROS levels and inhibited tight junction disruption, determined by zona occludens-1 immunostaining, in mice [50]. Lutein intake increased macular pigment optical density and visual contrast sensitivity in 90 patients with atrophic age-related macular degeneration [51], suggesting the lutein intake-mediated improvement in visual function.

Human clinical trials reported that individuals receiving lutein/zeaxanthin supplements experienced less vision loss than the controls [52]. Ma et al. [53] showed that a 12-week lutein supplementation improved visual function in healthy subjects exposed to long-term computer display light. These studies show that a high intake of lutein may have beneficial effects on visual performance.

Cataracts occur due to the loss of lens transparency caused by the aggregation of lens crystallins [54]. The risk factors attributed to the onset of cataracts include aging, diabetes, exposure to UV light, hypertension, and oxidative stress [55]. ROS cause cross-linking and degradation of lens proteins, thereby initiating cataractogenesis [56]. Padmanabha and Vallikannan [57] showed that eicosapentaenoic acid and docosahexaenoic acid increased the anti-cataract activity of lutein. Lutein decreased the serum and lens malondialdehyde levels, and the serum eicosanoids (PGE_2_, leukotriene B_4_, and leukotriene C_4_), C-reactive protein, and cytokines (TNF-α, IL1-β, and MCP-1), but increased the activities of antioxidant enzymes catalase, superoxide dismutase (SOD), and glutathione peroxidase in rats. They suggested that therapy with lutein, eicosapentaenoic acid, and docosahexaenoic acid for regulation of oxidative stress and inflammation to counter cataracts may be more effective.

**Figure 2:**
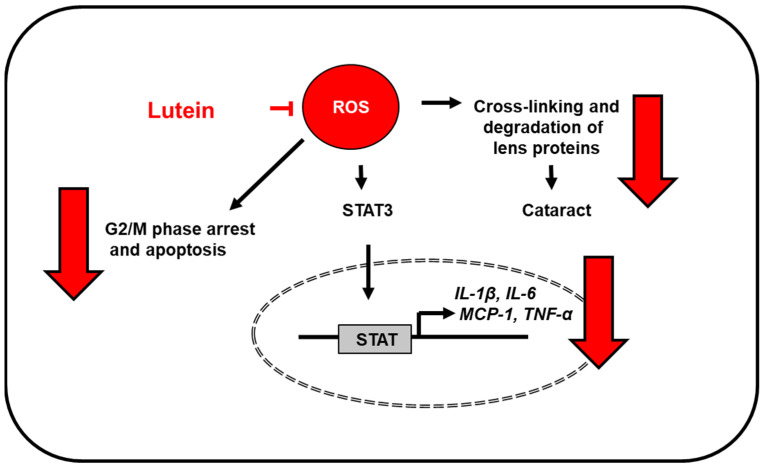
The proposed mechanism by which lutein inhibits oxidative stress-induced inflammatory responses in the eye. ROS levels increase in aged retina and lipopolysaccharide-stimulated retinal pigment epithelial cells. Lutein reduces ROS levels and inhibits ROS-mediated activation of STAT3 [7] and the expression of inflammatory mediators (IL-1β, IL-6, MCP-1, TNF-α) [7,49,57]. Thus, it prevents age-related macular degeneration. In addition, lutein prevents oxidative stress-mediated G2/M arrest and apoptosis in retinal pigmental epithelial cells [48] and cross-linking and degradation of lens proteins which prevents cataractogenesis [56]. IL, interleukin; MCP-1; monocyte chemoattratant protein-1; ROS, reactive oxygen species; STAT, signal transducer and activator of transcription; TNF-α, tumor necrosis factor-α.

The biological role of lutein in the retina and lens has not yet been well elucidated, but these findings suggest that dietary lutein supplementation may be beneficial for preventing age-related macular degeneration and other eye diseases by reducing oxidative stress. The proposed mechanism by which lutein inhibits oxidative stress-induced inflammatory responses in the eye is shown in Figure 2.

### 2.3. Osteoporosis

**Figure 3:**
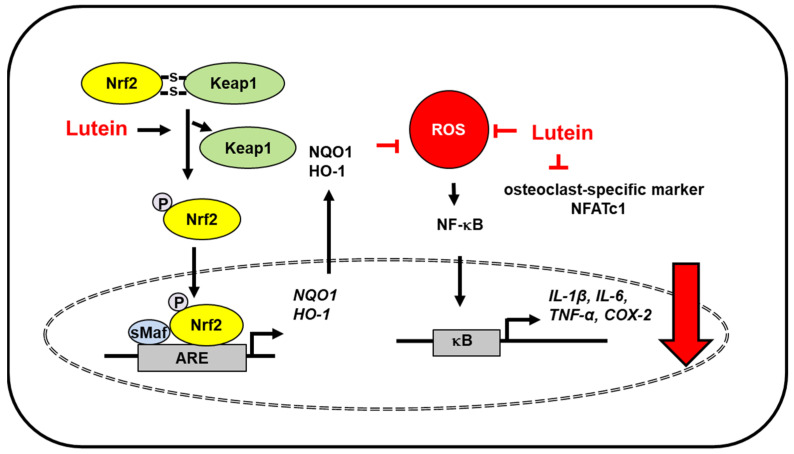
The proposed mechanism by which lutein inhibits oxidative stress-induced inflammatory responses in bone. ROS levels increase in monosodium iodoacetate-induced osteoarthritis in primary chondrocyte cells and femur tissues of ovariectomized rats (osteoporosis model). Lutein reduces ROS levels and inhibits ROS-mediated activation of NF-kB and the expression of inflammatory mediators (IL-1β, IL-6, TNF-α, COX-2). Moreover, lutein increases dissociation of Keap1 from Nfr2/Keap1 complex and thus, promotes nuclear translocation of Nrf2, which forms a heterodimer with sMaf protein and binds to the regulatory region of DNA called ARE. It induces the expression of Nrf2- target antioxidant genes (HO-1, NQO1). These antioxidant enzymes reduce intracellular ROS levels, which suppresses inflammatory responses [58,59]. In addition, lutein inhibits osteoclast-specific marker NFATc1 in the bone of ovariectomized rats [59]. Thus, lutein prevents oxidative stress-mediated osteoarthritis and bone deterioration. ARE, antioxidant response element; COX-2, cyclooxygenase-2; HO-1, hemeoxygenase-1; IL, interleukin; Keap1, kelch like ECH associated protein 1; NF−κB, nuclear factor-κB; Nrf2, nuclear factor erythroid 2–related factor 2; NQO-1, NAD(P)H:quinone oxidoreductase 1; NFATc1, nuclear factor of activated T cells 1; ROS, reactive oxygen species; sMaf, small Maf; TNF-α, tumor necrosis factor-α.

Due to its anti-inflammatory effects, lutein is expected to have bone-protective properties. Lutein treatment inhibited inflammatory proteins (NF-κB, COX-2) and pro-inflammatory cytokines (IL-6, TNF-α, IL-1β) in monosodium iodoacetate-induced osteoarthritis in primary chondrocyte cells. Lutein treatment prevented apoptosis of chondrocytes and enhanced expression of Nrf2 and its downstream target antioxidant genes HO-1 and NQO-1 in monosodium iodoacetate-treated cells. This study shows that lutein has cytoprotective effects against osteoarthritis through Nrf2 activation-mediated modulation of oxidative stress and inflammation [58] (Figure 3).

Osteoporosis is caused by hormonal imbalance and increased redox signaling, which induce bone deterioration. Lutein supplementation in ovariectomized rats decreased oxidative stress owing to its antioxidant protection. Lutein protected ovariectomized rats from osteoporosis by reducing lipid peroxidation, inhibiting NF-κB activation, and reducing the levels of inflammatory cytokines (TNF-α, IL-6, IL-8) and osteoclast-specific marker [nuclear factor of activated T cells 1 (NFATc1)]. Further, lutein upregulated Nrf2-driven antioxidant gene expression (HO-1, NQO1) in ovariectomized rats [59] (Figure 3).

Lutein increased the formation of mineralized bone nodules by upregulating bone morphogenetic protein 2 expression and downregulating sclerostin expression in osteoblast cultures [60]. Bone morphogenetic protein 2 plays a critical role in osteoblast differentiation and new bone formation. Sclerostin has anti-anabolic effects on bone formation. IL-1-induced osteoclast differentiation and bone resorption were suppressed by lutein [61]. Four-week supplementation with lutein increased the femoral bone mass in growing mice by stimulating bone formation and suppressing bone resorption [61].

Epidemiological studies have found a positive correlation between bone mass and carotenoid intake [62]. Dietary total carotenoids, α-, β-carotene, and lutein, were associated with a low risk of hip fracture in men [63]. Since total oxidative/anti-oxidative status is related to bone mineral density in osteoporosis [64], the intake of a lutein-rich diet can improve bone mineral status and may reduce the risk of osteoporosis and fracture. In general, lutein may be beneficial to bone health.

### 2.4. Cardiovascular Diseases

Lutein has been introduced as a potential candidate for atheroprotection. Dwyer et al. [65] investigated the effect of lutein on the development of early atherosclerosis using epidemiological study, in vitro study, and a mouse model. An epidemiological study showed that subjects with the highest level of serum lutein (0.42 μmol/L) showed 80% lesser arterial wall thickening than those with the lowest quintile of serum lutein (0.15 μmol/L). In a study on monocyte migration in a co-culture model of human intima, lutein inhibited low-density lipoprotein-induced migration of monocytes in a dose-dependent manner. Lutein supplementation reduced atherosclerotic lesion formation in model mice [65]. According to a study conducted in Beijing, which comprised 125 subjects with early atherosclerosis and 107 controls aged 45–68 years, serum levels of lutein were significantly lower in cases of early arteriosclerosis than in controls. Serum lutein was observed to be inversely related to carotid intima-media thickness, an index of arteriosclerosis. However, there was no significant difference in zeaxanthin and β-carotene levels between the cases and controls [66].

Inflammation induces multiple risk factors for atherosclerosis and its complications [67]. The development of atherosclerosis lesions is initiated by oxidized low-density lipoprotein, leading to endothelial dysfunction and increased monocyte and chemokine levels. Subsequently, increased levels of cytokines and chemokines maintain and amplify the inflammatory responses [68]. The extent of inflammatory infiltrates and their strategic location within the protective fiber were related to plaque rupture or thrombosis in patients with atherosclerosis [69]. Speicific inflammatory mediators such as adhesion meolecules and chemoattractant proteins are involved in the pathogenesis of atherosclerosis [70,71].

**Figure 4:**
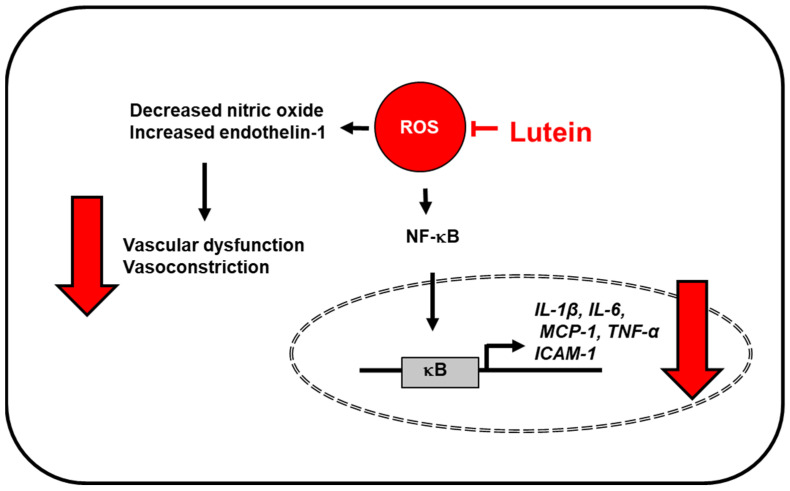
The proposed mechanism by which lutein inhibits oxidative stress-induced inflammatory responses in vascular endothelial cells. ROS levels increase in vascular endothelial cells exposed to high concentrations of homocysteine (atherosclerosis model) or lipopolysaccharide. Lutein reduces ROS levels and inhibits ROS-associated activation of NF-kB and expression of inflammatory mediators (IL-1β, IL-6, MCP-1, TNF-α, ICAM-1) in endothelial cells [71,76]. Moreover, lutein inhibits ROS-induced vascular dysfunction (decreased nitric oxide and increased endothelin-1), and thus, prevents vasoconstriction [71]. ICAM-1, intercellular adhesion molecule 1; IL, interleukin; MCP-1; monocyte chemoattratant protein-1; NF−κB, nuclear factor-κB; ROS, reactive oxygen species; TNF-α, tumor necrosis factor-α.

Oxidative stress is also an important factor of atherosclerosis-associated endothelial injury and inflammation. Wang et al. [71] showed the effect of lutein intervention on hyperhomocysteinemia-mediated atherosclerosis. This study reported that hyperhomocysteinemia decreased vasodilator nitric oxide (NO) level and increased endothelin-1 level, which is associated with vascular endothelial dysfunction, but these levels were reversed by lutein. Lutein intervention also inhibited hyperhomocysteinemia-induced oxidative stress and downregulated inflammatory factors such as NF-κB p65, TNF-α, and intercellular adhesion molecule 1 [71] (Figure 4). As hyperhomocysteinemia induces oxidative stress and endothelial dysfunction, it can be associated with cardiovascular disease [72,73]. In TNF-α-treated vascular endothelial cells, lutein treatments improved basic endothelial function with increased NO and decreased release of endothelin-1 through inhibition of NF-κB signaling [74]. These results supported the effect of lutein on vascular structure and function to prevent atherosclerosis development and progression.

Endothelial function is modulated by vasodilators and vasoconstrictors. Vasodilator NO deficiency results in general vasoconstriction and hypertension. Lutein prevents hypertension through various pathways, including its influence on NO synthesis and enhancement of antioxidant properties [75].

Lutein supplements reduced the levels of serum inflammatory cytokines (IL-6, MCP-1), low-density lipoprotein, and triglyceride, which play important roles in the development of early atherosclerosis in patients [70]. Accumulating evidence also suggests a protective effect of lutein on cardiovascular disease and coronary heart disease. Most patients with coronary artery disease have chronic low-grade inflammation. Clinical findings have reported an inverse association between serum levels of lutein and IL-6 in patients with stable angina. When peripheral blood mononuclear cells from patients with coronary artery disease were pretreated with lutein, followed by treatment of lipopolysaccharide, it lowered lipopolysaccharide-induced secretion of IL-6, IL-1β, and TNF, and downregulated IL-6, IL-1β, and TNF mRNA expression in a dose-dependent manner [76] (Figure 4). Among carotenoids, including oxygenated carotenoids (lutein, zeaxanthin, β-cryptoxanthin) and hydrocarbon carotenoids (α-carotene, β-carotene, lycopene), serum levels of oxygenated carotenoids were reduced in patients with coronary artery disease, which was correlated with a low level of high-density lipoprotein that increases the risk of coronary artery disease [77]. These results support the potential anti-inflammatory effects of lutein in patients with coronary artery disease.

Individuals with a history of atherosclerosis showed higher blood concentrations of complement factors C3 and C3a than subjects who have no such a history. C3 forms a membrane attack complex through an alternate complement pathway, creating a hole or pore in the membrane that can kill pathogens or host cells. Lutein has been shown to reduce the levels of plasma complement factors, including membrane attack complex. Thus, lutein may prevent or reduce tissue oxidation and prevent activation of damaging complement factors in the blood, leading to atheroprotection and cardiometabolic health [78].

### 2.5. Skin Diseases

Lutein reduced ROS formation following ultraviolet (UV) irradiation, thus prevented the photo-oxidative damage and reversed contact hypersensitivity reactions which were suppressed by UVB in mice [79]. A human study showed that oral supplementation of lutein and zeaxanthin improved overall skin tone and induced skin-lightening effects, which may be due to their antioxidant activities [80]. UV radiation and UVB radiation stimulate immunosuppressive and oxidative stress-inducing mechanisms that contribute to skin cancer, photodermatoses, sunburn, and photoaging [81,82]. In a human study, lutein supplementation (lutein soft gel capsules containing 10 mg free lutein stabilized by 10% carnosic acid for 12 weeks) reduced the mRNA expression of intercellular adhesion molecule 1 and metalloproteinase-1, which are indicators of photodermatoses and photoaging [83]. These studies indicate that lutein has a protective effect against UV-induced skin damage. Dietary lutein provided protection against skin swelling and hyperplasia caused by UV exposure in hairless mice [84]. Furthermore, lutein intake inhibited UVB-induced skin swelling, reversed the inhibition of contact hypersensitivity, and decreased ROS generation following UV radiation exposure in mice [79]. These results suggest that lutein reduces UV-induced inflammation and immunosuppression. In addition, lutein inhibits transient receptor potential ankyrin 1 activation-induced neutrophil accumulation, leading to suppression of skin inflammation [10].

Palombo et al. [85] demonstrated that 12-week Oral administration of lutein (10 mg/day) and zeaxanthin (0.6 mg/day) reduced skin lipid peroxidation (malondialdehyde level) and exhibited photoprotective activity following UV irradiation. Balic and Mokos [86] showed that β-carotene, lycopene, lutein, and astaxanthin exhibit photoprotective effects by direct light-absorbing properties, scavenging ROS, and/or suppressing inflammation. They demonstrated that human subjects with a carotenoid-rich diet showed decreased sensitivity to UV radiation-induced erythema (photoprotective effects on skin) and enhanced skin elasticity and hydration, skin texture, wrinkles, and age spots (anti-aging effect on skin). Thus, dietary intake of lutein is important for maintaining skin health and functions. 

### 2.6. Liver Injury

Alcoholic liver disease leads to steatosis, steatohepatitis, cirrhosis, and hepatocellular carcinoma. Alcohol is metabolized to toxic metabolites that cause redox imbalance [87]. Oxidative stress mediates inflammatory responses of hepatic cells, such as disturbances in calcium homeostasis, activation of mitogen-activated protein kinases and redox-sensitive transcription factors (such as NF-κB), and apoptosis, leading to alcohol-induced liver injury [88,89,90]. Therefore, reducing oxidative stress is expected to ameliorate alcohol-induced liver damage.

**Figure 5:**
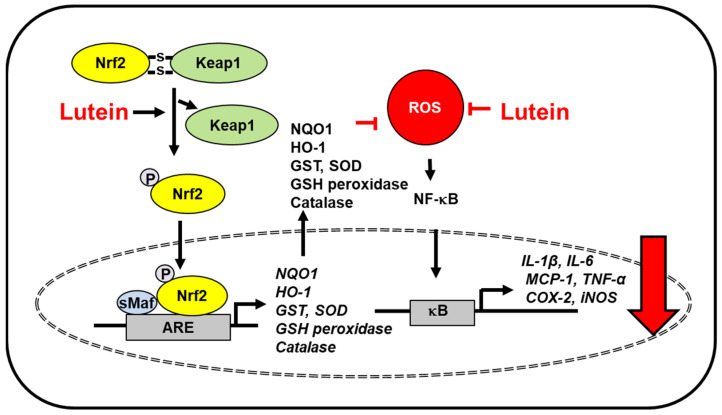
The proposed mechanism by which lutein inhibits oxidative stress-induced inflammatory responses in the liver. ROS levels increase in hepatic tissues exposed to hepatotoxins such as ethanol or arsenic pollutant. Lutein reduces ROS levels and inhibits ROS-mediated activation of NF-kB and the expression of inflammatory mediators (IL-1β, IL-6, MCP-1, TNF-α, COX-2, iNOS). Moreover, lutein increases dissociation of Keap1 from Nfr2/Keap1 complex and thus, promotes nuclear translocation of Nrf2, which forms a heterodimer with sMaf protein and binds to the regulatory region of DNA called ARE. It induces the expression of Nrf2- target antioxidant genes (HO-1, NQO1, GST, SOD, glutathione peroxidase, catalase). These antioxidant enzymes reduce intracellular ROS levels, which suppresses inflammatory responses [92,95]. Thus, lutein prevents oxidative stress-mediated hepatotoxicity. ARE, antioxidant response element; COX-2, cyclooxygenase-2; GSH, glutathione; GST, glutathione-s-transferase; HO-1, hemeoxygenase-1; IL, interleukin; iNOS, inducible nitric oxide synthase; Keap1, kelch like ECH associated protein 1; NF−κB, nuclear factor-κB; Nrf2, nuclear factor erythroid 2–related factor 2; NQO-1, NAD(P)H:quinone oxidoreductase 1; NFATc1, nuclear factor of activated T cells 1; ROS, reactive oxygen species; sMaf, small Maf; SOD, superoxide dismutase; TNF-α, tumor necrosis factor-α.

Lutein showed ROS scavenging and protection of the liver from hepatotoxins such as carbon tetrachloride, ethanol, and paracetamol in rats. Lutein administration reduced lipid peroxidation and conjugated dienes and hydroperoxides in the liver tissue in paracetamol-treated rats and increased the levels of antioxidant enzymes, such as superoxide dismutase, catalase, glutathione peroxidase, and glutathione during alcohol- and carbon tetrachloride-induced liver toxicity [91]. Lutein (40 mg/kg body weight), gavaged 30 min before ethanol treatment, decreased the levels of oxidative stress markers (ROS, lipid peroxidation, protein carbonyls, and sulfhydryls content), liver markers (aspartate aminotransferase, alanine aminotransferase, lactate dehydrogenase, and alkaline phosphatase), inflammatory proteins (NF-κB, COX-2, iNOS), and inflammatory cytokines (TNF-α, MCP-1, IL-1β, IL-6), but increased the Nrf2 levels and activities of antioxidant enzymes [catalase, glutathione peroxidase, glutathione, glutathione-s-transferase (GST)] in rats [92] (Figure 5).

Kim et al. [93] reported that in hypercholesterolemic guinea pigs, 12 week-supplementation of lutein [0.1 g lutein/100 g high cholesterol diets (0.25% cholesterol)] reduced hepatic free cholesterol and hepatic TNF-α levels by attenuating the DNA-binding activity of NF-κB, compared with the control group. Mai et al. [94] showed that lutein treatment (40 mg lutein/kg body weight/day) decreased iNOS levels in the liver of mice with D-galactose-induced liver injury. A mouse model showed that lutein treatment alleviated arsenic pollutant-induced hepatotoxicity by increasing the levels of Nrf2 signaling-related antioxidant enzymes (NQO1, HO-1, and GST) and reducing ROS and malondialdehyde levels in the liver [95]. Thus, lutein may reduce oxidative stress and inflammatory responses by activating Nrf2 signaling and inducing Nrf2-target antioxidant enzymes in the liver, thereby protecting the liver against hepatotoxins (Figure 5).

### 2.7. Obesity

Obesity is caused by excess intake of energy-dense foods and low physical activity, and it is a major risk factor for chronic diseases such as type 2 diabetes mellitus, hypertension, cardiovascular diseases, and cancer [96,97]. The levels of oxidative stress and inflammatory factors correspond to the amount of adipose tissue [98]. In particular, visceral fat is linked with the risk of obesity-associated diseases because it is related to insulin resistance (IR) and increased the levels of inflammatory mediators MCP-1, IL-6, TNF-α, and C-reactive protein [99,100].

Serum lutein and zeaxanthin levels were observed to be inversely related to serum CRP concentrations [101]. Interestingly, serum levels of lutein and zeaxanthin were found higher in Mexican American and African American children and adolescents than in White American children and adolescents, based on the data from the U.S. NHANES III (1988–1994).

Gopal et al. [102] showed that the accumulation of lipid droplets was significantly decreased in lutein-treated 3T3-L1 cells. This study found that lutein downregulated CCAAT/enhancer-binding protein-α (CEBP-α) and peroxisome proliferator-activated receptor-γ (PPAR-γ) during the early stage of adipocyte differentiation, which repressed the phosphorylation of protein kinase B and ERK. Blocking the initial stages of differentiation reduced mature adipocyte development and lipid accumulation.

Several studies have demonstrated a negative association between dietary lutein and serum lutein levels and adiposity [103,104]. Increased adiposity may also lead to inefficient delivery of lutein to the macula because adipose tissue acts as a sink for lutein [105,106]. Johnson [106] suggested that increased body fat induced oxidative destruction of endogenous lutein and changed lipoprotein profile, affecting the circulatory delivery of lutein to the macular of the eye.

In addition, the possible effects of lutein and zeaxanthin administration on lipid profile, oxidative stress, and inflammation pathways were investigated in a rodent model of high-fat diet-induced obesity [107]. Lutein and zeaxanthin supplementation reduced the levels of free fatty acids and oxidative stress markers (increased malondialdehyde levels and decreased antioxidant enzyme activities) in the retina of rats receiving a high-fat diet. These supplementations reduced the levels of vascular endothelial growth factor, NF-κB, and intercellular adhesion molecule 1 and enhanced Nrf2 and HO-1 protein expression in retinal tissues, which may have contributed to the alleviation of high fat diet-induced retinal injury. Collectively, lutein may be an effective treatment for retinal damage in obesity.

### 2.8. Colon Diseases

Ulcerative colitis is a long-term inflammatory condition of the colon and rectum [108]. Rana et al. [109] demonstrated that erythrocytes of patients with ulcerative colitis from northern India showed higher malondialdehyde levels but lower glutathione levels than healthy controls. In mice with dextran sulfate sodium-induced ulcerative colitis, lutein was supplemented in the form of dry hydroalcoholic extract of Tagetes erecta flowers (DHETE), and it reduced myeloperoxidase activity and levels of TNF and IL-6 [110]. Moreover, the extract reversed the reduction of glutathione levels and catalase activity and normalized the SOD and GST levels in the colon tissues. DHETE (300 mg/kg) prevented dextran sulfate sodium-induced weight loss, colon shortening, and morphological changes in rats. Further, lutein concentration in the DHETE was estimated at 8.2%. These studies showed the involvement of oxidative stress in the pathogenesis of ulcerative colitis, which was reversed by lutein treatment.

Rumi et al. [111] demonstrated lower levels of lutein and zeaxanthin in patients with Crohn’s disease than in healthy subjects. Thus, intake of lutein and zeaxanthin may be beneficial for preventing the progression of Crohn’s disease. Overall, lutein is expected to be a potential treatment for gastrointestinal disorders; however, large-scale human studies are needed to support the role of lutein in gastrointestinal protection in humans.

### 2.9. Diabetes

In the serum and retina of the diabetic population, low levels of lutein have been observed. Sahli et al. [112] found that a lutein-rich diet protects against the development of diabetic retinopathy in individuals with diabetes enrolled in a population-based cohort study. The protective effects of lutein on the retina have been reported in various studies. Wang et al. [113] showed that long-term lutein supplementation decreased retinal inflammation and functional deficits in early diabetic retinopathy using the genetic model for diabetic retinopathy. Another study examined the protective effect of lutein on hyperglycemia-mediated oxidative stress and antioxidant defense activity in retinal pigment epithelial cells [114]. This study reported that lutein treatment reduced ROS levels and reversed down-regulation of Nrf2 and antioxidant enzymes, SOD 2, HO-1, and catalase in APRE-19 cells. Lutein-induced activation of Nrf2 was linked to increased activation of regulatory proteins ERK and protein kinase B. These findings demonstrated that increasing concentration of lutein in the retina could protect the retina from diabetes-induced retinopathy. A systematic review and meta-analysis [115] showed that lutein might be beneficial for atherosclerosis and inflammatory markers, but there were inconsistent associations with blood pressure, adiposity, insulin resistance, and blood lipids. Although lutein can be a potential treatment for diabetes with its antioxidant properties, more preclinical and clinical studies are examined to confirm these above findings.

## 3. Conclusions

Due to its free radical scavenging activity, lutein reduces oxidative stress and inflammatory responses in various organs. Inflammatory stimuli and environmental stress, including UV light, may increase the production of ROS. Lutein reduces ROS levels and inhibits ROS-mediated activation of NF-kB and STAT3, and thus the expression of inflammatory mediators (IL-1β, IL-6, MCP-1, TNF-α, COX-2, iNOS). Lutein promotes Nrf2 activation and the expression of Nrf2- target antioxidant genes (HO-1, NQO1, GST, SOD, glutathione peroxidase, catalase) to reduce ROS levels. Since lutein reduces oxidative stress, it maintains the levels of endogenous antioxidants such as glutathione. The inhibitory effects on inflammatory signaling pathways and enhanced antioxidant activities of lutein may be the underlying mechanisms of protection against inflammation-related diseases.

Studies on lipopolysaccharide-stimulated microglia and high glucose-treated retinal pigment epithelial cells, lutein activates ERK, which may phosphorylate Nrf2 and Nrf2 activation induces production of Nrf2-driven antioxidant enzymes.

Lutein supplement reduces the levels of serum low-density lipoprotein and triglyceride, which play an important role in the development of early atherosclerosis in patients. In addition, lutein has light-absorbing and ROS-scavenging properties, which contribute to protection against UV light-induced skin damage; it suppresses transient receptor potential ankyrin 1-induced skin inflammation. Lutein reduces lipid droplet formation and downregulates CEBP-α and PPAR-γ during the early stage of adipocyte differentiation, which represses obesity-related inflammation. The effects of lutein on inflammatory responses in experimental models and epidemiological studies were summarized in Table 1 and Table 2.

In conclusion, lutein downregulates redox-sensitive inflammatory signaling pathways and inhibits the induction of inflammatory mediators. Therefore, it may prevent various inflammatory diseases, including neurodegenerative disorders, eye diseases including diabetic retinopathy, osteoporosis, cardiovascular diseases, skin diseases, liver injury, obesity, and colon diseases. Moreover, lutein exhibits tissue-specific actions, such as regulating lipid profiles in cardiovascular diseases, adipocyte differentiation in obesity, and skin function. Therefore, the consumption of lutein-rich foods may be beneficial in preventing oxidative stress-induced inflammatory diseases.

## Figures and Tables

**Table 1:** The effects of lutein on inflammatory responses in experimental models.

Diseases	Experimental Model	Lutein Dose	Key Findings	Ref.
Neurodegenerative disorders	rats with severe traumatic brain injury	40, 80, 160 mg/kg body weight (BW)	-suppressed IL-1β, IL-6, and monocyte chemoattractant protein (MCP)-1 expression -reduced serum reactive oxygen species (ROS) levels -downregulated the expression of nuclear factor-κΒ (NF-κB) p65, and cyclooxygenase (COX) -2 -upregulated nuclear factor erythroid 2–related factor 2 (Nrf2) and endothelin-1 protein levels	[37]
	LPS-induced neuroinflammation in mouse microglial cells	50 μM	-inhibited inducible nitric oxide synthase (iNOS) and COX-2 expression -inhibited TNF-α, IL-1β, and nitric oxide (NO) production -suppressed lipopolysaccharide-induced NF-κB activation -decrease of Keap1 and activation of Nrf2, and subsequent upregulation of heme oxygenase(HO)-1 and NAD(P)H: quinone oxidoreductase 1 (NQO1) in the presence or absence of LPS -induced activation of extracellular signal-regulated kinase (ERK), which was linked to Nrf2 activation	[38]
	acute inflammation-induced sensitization of nociceptive processing in rats	10 mg/kg BW	-decreased in the inflammation-induced mean times of face grooming and the thickness of inflammation-induced edema in whisker pads -decreased numbers of c-Fos-positive neurons in both spinal trigeminal nucleus caudalis and upper cervical dorsal horn neurons	[42]
Eye diseases	H_2_O_2_ stress-induced acute retinal pigment epithelial cells	0, 1, 5, 10 and 15 µM	-increased cell viability, and decreased apoptosis and ROS levels -reversed the increased proportion of cells in the G2/M phase in a concentration-dependent manner -attenuated cell cycle arrest in G2/M phase by activating cyclin-dependent kinase 1 and cell division cycle 25C, and decreasing cyclin B1	[48]
	Lipopolysaccharide-stimulated human retinal pigment epithelial cells	1, 10 μM	-reduced expression of IL-6 and IL-8 dose-dependently	[49]
	sodium selenite-induced cataract in male Wistar rat pups	1.3 μmol/kg BW	-lutein + eicosahexaenoic acid (EPA) + docosahexaenoic acid (DHA) exhibited the highest cataract prevention -pups had the highest amount of lutein in the lens with lutein + EPA + DHA	[57]
Osteoporosis	monosodium iodoacetate-induced osteoarthritis in primary chondrocyte cells	0.5, 1, 5 and 10 μM	-increased the cell viability of chondrocytes -downregulated inflammatory proteins (NF-κB, COX-2) and pro-inflammatory cytokines (IL-6, TNF-α, IL-1β) -reduced monosodium iodoacetate-induced apoptosis through downregulating the caspase-3 activity	[58]
	ovariectomized rats (osteoporosis model)	50 mg/kg BW	-decreased the lipid peroxidation and ROS levels -activated Nrf2-driven antioxidant gene expression (HO-1, NQO1) -downregulated osteoclast-specific marker [nuclear factor of activated T cells 1 (NFATc1)] expression	[59]
	mouse bone marrow cells and osteoblastic cells	3, 10, 30 μM	-inhibited IL-1-induced osteoclast differentiation and bone resorption -enhanced the formation of mineralized bone nodules by increasing bone morphogenetic protein 2 expression and inhibiting sclerostin expression	[60]
	newborn and 5- and 6-week-old ddy mice	66 mg/kg BW	-enhanced the femoral bone mass in growing male mice in vivo	[61]
	bone marrow cells isolated from 6-week-old mice and co-cultured with primary osteoblast cells	3, 10 µM	-stimulated bone formation and suppressed bone resorption in vitro -regulated Receptor activator of NF-kΒ ligand (RANKL)-dependent osteoclast formation in vitro	[61]
Cardiovascular diseases	a co-culture model of the artery wall formed from endothelial and smooth muscle cells from human aortas	0.1, 1.0, 10, and 100 nmol/L	-inhibited low-density lipoprotein-induced migration in a dose-dependent manner in vitro	[65]
	apoE-null mice	0.2% by chow weight	-reduced atherosclerotic lesion size in the aortic arch in apoE-null mice in vivo -reduced plasma very low-density lipoprotein+ intermediate-density lipoprotein in vivo	[65]
	hyperhomocysteinemia rat	20 mg/kg BW	-increased serum levels of superoxide dismutase (SOD) and glutathione peroxidase -downregulated the expression of NF-kΒ and intercellular adhesion molecule-1 -increased the serum NO level and decreased endothelin-1 content	[71]
	two cultured endothelial cell models (EA.hy926 and human umbilical vein endothelial cells)	0.3 µmol/L	-decreased TNF-α -induced leukocytes adhesion, expression of intercellular adhesion molecule-1, and vascular cell adhesion molecule 1 -attenuated leukocytes adhesion to endothelial cells	[74]
Skin diseases	female C3H/HeJ mice	0.04, 0.4% lutein of 100 g diet	- inhibited UVB-induced skin swelling and reversed the inhibition of contact hypersensitivity) -reduced local UVB-radiation-induced immune suppression -reduced ROS generation in murine skin	[79]
	female hairless SKh-1 mice	0.4%, 0.04% lutein and zeaxanthin of 100 g diet	-suppressed UVB-induced skin inflammation -decreased the number of UVB-induced apoptotic keratinocytes -decreased the number of UVB-induced proliferating cell nuclear antigen-positive cells in murine skin -reduced UVB-induced increase in bromodeoxyuridine incorporation into basal epidermal keratinocytes	[84]
Liver Injury	rats with alcohol-induced liver injury	40 mg/kg BW	- reduced hepatic tissue levels of oxidative stress markers (ROS levels, lipid peroxidation, protein carbonyls, and sulfhydryls content), inflammatory cytokines (TNF-α, MCP-1, IL-1β, IL-6), and inflammatory proteins (NF-κB, COX-2, iNOS) -reduced serum levels of liver markers (aspartate aminotransferase, alanine aminotransferase, lactate dehydrogenase, and alkaline phosphatase -increased tissues levels of Nrf2 and activities of antioxidant enzymes (catalase, glutathione peroxidase, glutathione, glutathione-*s*-transferase)	[92]
	hypercholesterolemic diet-fed guinea pigs	0.1 g lutein/100 g high cholesterol diets (0.25% cholesterol)	-reduced hepatic free cholesterol -reduced hepatic malondialdehyde level -reduced hepatic TNF-α and NF-κB DNA binding activity	[93]
	Kunming strain mice received D-galactose-induced oxidative stress	10, 40 mg/kg BW	-decreased ROS contents in liver tissues -increased the activity of Na(+)- K(+)-ATPase and the activity of Ca(2+)-ATPase in liver tissues -decreased the activities of total NO synthase and iNOS and the content of NO in liver tissues -increased HO-1 mRNA, but decreased Toll-like receptor 4 mRNA expression in liver tissues	[94]
	arsenic trioxide-induced liver damage in Kunming mice	40 mg/kg BW	-reduced ROS and malondialdehyde in liver tissues -increased mRNA and protein expression of Nrf2 signaling related genes [Nrf2, HO-1, NQO1, and glutathion-*s*-transferase] -improved hepatic function comparing with arsenic trioxide group	[95]
Obesity	3T3-L1 adipocyte model	1, 5, 10 μM	-decreased the numbers and sizes of the lipid droplets -reduced triglyceride content in a concentration-dependent manner -suppressed the expression of transcription factors [CCAAT-enhancer-binding protein (CEBP)-α and peroxisome proliferator-activated receptor (PPAR)-γ] and associated adipogenic markers (fatty acid synthase, fatty acid-binding protein 4, and stearoyl-CoA desaturase 1) -blocked the process of adipogenesis at the early stage of adipocyte differentiation -delayed cell cycle progression with increased cell count at G0/G1 phase through regulating the levels of cyclin D and E, cyclin-dependent kinase 4, and cyclin-dependent kinase 2	[102]
	high fat-diet induced obesity rats	100 mg/kg	-decreased free fatty acid levels and oxidative damage by reducing MDA levels -improved activities of SOD, catalase, and glutathione peroxidase -decreased levels of vascular endothelial growth factor, intracellular adhesion molecule-1, iNOS, and NF-κB -increased Nrf-2 and HO-1 levels	[107]
Colon diseases	LPS-induced murine intestinal epithelial cells	100 μg/mL	-reduced ROS and NO production in lipopolysaccharide-stimulated IEC-6 cells	[110]
	dextran sulfate sodium-treated mice	30, 100, 300 mg/kg	-attenuated weight loss, disease activity index, colon shortening, and histopathological changes in dextran sulfate sodium-treated mice -decreased myeloperoxidase activity as well as TNF and IL-6 levels -increased glutathione levels and catalase activity -normalized SOD and glutathione-*s*-transferase activities	[110]
Diabetes	$\mathrm{Ins}2^{\mathrm{Akita}/+}$ mice (model of early diabetic retinopathy)	2.1, 4.2, 8.4 mg/kg in drinking water	-suppressed microglial reactivity -reduced the upregulation of vascular endothelial growth factor -attenuated retinal vascular leakage -protected retinas from functional impairment	[113]
	retinal pigment epithelial cells	0.5, 1 μM	-blocked high glucose-mediated elevation of intracellular ROS, protein carbonyl, and malondialdehyde content -reversed down-regulation of a redox-sensitive transcription factor, Nrf2, and antioxidant enzymes, SOD2, HO-1, and catalase	[114]

**Table 2:** The effects of lutein on inflammatory responses in epidemiological studies.

Diseases	Epidemiological Study	Study Design	Key Findings	Ref.
Neurodegenerative disorders	cross-sectional study, the Irish longitudinal study on aging	4076 individuals, aged 50 and older	-higher plasma lutein and zeaxanthin were associated with better composite scores across the domains of global cognition, memory, and executive function	[31]
	case-control study	25 patients with mild cognitive impairment, 63 Alzheimer’s disease patients, and 53 controls, mean age of 75.8	-plasma antioxidants (vitamin C, uric acid, vitamin A, vitamin E, carotenoids; lutein, zeaxanthin, β-cryptoxanthin, lycopene, β-carotene, α-carotene) are depleted in patients with mild cognitive impairment and Alzheimer’s disease -activities of plasma SOD and plasma glutathione peroxidase decreased in patients with mild cognitive impairment and Alzheimer’s disease	[33]
	cross-sectional study in the NHANES III database and the NHANES III linked mortality file	total of 6958 participants, aged 50 and older	-high serum levels of lutein + zeaxanthin at baseline were associated with a lower risk of Alzheimer’s disease mortality	[35]
Eye diseases	cross-sectional study in the NHANES III (third national health and nutrition examination survey)	8222 persons, aged 40 and older	-higher dietary intakes of lutein and zeaxanthin were related to lower odds for pigmentary abnormalities and one sign of early age-related maculopathy (determined by serum carotenoids levels, food frequency questionnaire, and retinal photographs)	[44]
	randomized double-masked placebo-controlled clinical trial-oral treatment for 12 months	oral lutein supplementation	-treatment of oral preparation (lutein, zeaxanthin, vitamin C, vitamin E, copper, and zinc) for 12 months	[45]
	randomized double-masked placebo-controlled clinical trial-oral treatment for 12 months	433 adults, aged 55 and older with early age-related macular degeneration in at least one eye, 12 months follow up	-treatment group improved retinal function and increased macular pigment, as well as visual activity	[45]
	cross-sectional study in the age-related eye disease study (AREDS).	4519 AREDS participants, aged 60–80 years	-dietary lutein/zeaxanthin intake was inversely associated with neovascular age-related macular degeneration and large or extensive intermediate drusen	[55]
	a prospective, 12-month, randomized, double-masked, placebo-controlled trial	oral lutein supplementation 90 patients with atrophic age-related macular degeneration, mean age of 74.4 in lutein group, mean age of 76.1 in placebo group	-lutein (10 mg/d) for 12 months -lutein supplementation improved visual function -lutein supplementation increased eye macular pigment optical density	[56]
Osteoporosis	systematically review	women, aged 45 and older	-two cross-sectional analyses reported positive associations between food and vegetable intake and bone mineral density of the forearm, lumbar spine, or total hip	[62]
	cross-sectional study in the Singapore Chinese health study	63,257 men and women, aged 45–74 years, mean follow-up of 9.9 years	-consumption of lutein/zeaxanthin had a low risk of hip fracture risk among men (interviewed on lifestyle factors and medical history)	[63]
Cardiovascular diseases	The Los Angeles atherosclerosis study	269 women (aged 45–60) and 304 men (aged 40–60)	-an inverse association between plasma lutein and progression of carotid intima-media thickness	[65]
	case-control study from the Beijing atherosclerosis study	125 subjects with early atherosclerosis and 107 controls, aged 45–68 years	-serum lutein level was negatively correlated with carotid intima-media thickness	[66]
	cross-sectional study	134 patients with stable angina, aged 60–72	-plasma levels of lutein + zeaxanthin were inversely correlated with plasma levels of IL-6 in stable angina patients	[76]
	case-control study	39 patients with acute coronary syndrome and 50 patients with stable coronary artery disease, mean age of 59.3 50 controls, mean age of 60.8	-both patient groups had lower plasma levels of lutein + zeaxanthin -plasma levels of lutein+zeaxanthin were associated with the proportions of natural killer cells, but not with other lymphocytes, in blood (lutein and zeaxanthin may have a particular role in the immunological scenario of coronary artery disease)	[77]
	mini-review of revisit data in Toulouse and Belfast in 1992–1993	89 men and 82 women, aged 45–65 in 1992 102 salaried men and 109 women, aged 45–64 in 1993	-serum level of lutein was twice as high in Toulouse in Southern France as in Belfast in Northern Ireland in both men and women (incidence of coronary heart disease in Southern France was among the lowest in Europe and was higher in Northern Ireland) -high dietary intake of lutein reduced plasma concentrations of complement factors C3 and C3a as well as the membrane attack complex, the atherosclerosis indices	[78]
Skin diseases	a randomized, double-blind, placebo-controlled clinical trial	oral supplementation 50 healthy people, aged 18–45 with mild-to-moderate dry skin	-oral dietary supplement containing lutein (10 mg /d) and zeaxanthin isomers (2 mg /d) for 12 weeks- -treatment group improved overall skin tone and luminance values -treatment group increased mean minimal erythemal dose and the individual typological angle	[80]
	a randomized, double-Blind, placebo-controlled, 12-week clinical study	either oral, topical, or combined oral and topical administration 40 healthy women, aged 25–50	-either oral (lutein 10 mg/d, zeaxanthin 0.6 mg/d), topical (lutein 10 ppm/d, zeaxanthin 6 ppm/d), or mixed oral and topical administration of lutein and zeaxanthin -combined administration showed the highest effect on decreasing skin lipid peroxidation (reduced skin malondialdehyde level) -all three groups showed photoprotection against UV light irradiation-induced skin damage -all three groups improved skin elasticity and skin hydration	[85]
Obesity	cross-sectional study in NHANES III	total of 4231 males and nonpregnant females, aged 6–16	-serum level of high-density lipoprotein was directly related to serum levels of lutein + zeaxanthin -serum levels of C-reactive protein, an inflammation marker, were inversely related to serum levels of lutein + zeaxanthin	[101]
	cross-sectional study in NHANES III	8808 U.S. adults, aged 20 and older with and without the metabolic syndrome	-the age-adjusted concentration of lutein + zeaxanthin was lower in participants with metabolic syndrome than that of healthy control without the metabolic syndrome	[103]
	a population-based, cross-sectional study	374 men, aged 40–80	-higher lutein+zeaxanthin intakes were associated with lower subcutaneous fat mass	[104]
Diabetes	systemic review with meta analysis	71 relevant articles (including 387,569 participants)	-there was an inconsistent association with higher dietary lutein intake and insulin resistance	[115]
